# Genetic Polymorphisms of *Toll-like receptors 2* and *9* as Susceptibility Factors for the Development of Ankylosing Spondylitis and Psoriatic Arthritis

**DOI:** 10.1155/2019/1492092

**Published:** 2019-10-30

**Authors:** Camila F. Oliveira-Toré, Amarilis G. Moraes, Gabriela F. Martinez, Janisleya S. F. Neves, Luciana C. Macedo, Marco A. Rocha-Loures, Marília G. Quirino, Hugo V. Alves, Ana M. Sell, Jeane E. L. Visentainer

**Affiliations:** ^1^Post Graduation Program of Biosciences and Physiopathology, Department of Analysis Clinical and Biomedicine, Maringá State University, Parana, Brazil; ^2^Laboratory of Molecular Immunopathology, Department of Clinical Pathology, HC/UFPR, Curitiba, Parana, Brazil; ^3^Department of Medicine, Maringá State University, Parana, Brazil; ^4^Laboratory of Immunogenetics, Department of Basic Health Sciences, Maringá State University, Parana, Brazil

## Abstract

**Introduction:**

Ankylosing spondylitis (AS) and psoriatic arthritis (PsA) are classified as spondyloarthritis (SpA), a group of inflammatory rheumatic diseases with complex genetic etiology. Toll-like receptors (TLRs) have an important role in the mechanism of innate immunity and may influence inflammatory responses. Polymorphisms in *TLR* genes that lead to changes in these receptors or that interfere with the transcription rates of mRNA TLR may be involved in the chronic inflammatory immune response observed in SpA. Currently, there is a lack of studies associating genetic polymorphisms in *TLRs* and SpA.

**Objective:**

Therefore, this case-control study is aimed at analyzing the influence of the respective SNPs on *TLR2 rs5743708*, *TLR6 rs5743810*, and *TLR9 rs5743836* and *rs187084* in the immunopathogenesis of SpA.

**Methods:**

The polymorphisms genotyped by PCR-RFLP were *TLR2 rs5743708*, *TLR6 rs5743810*, and *TLR9 rs5743836* and *rs187084*. The *HLA-B*^∗^*27* was performed by PCR-SSP.

**Results:**

Logistic regression analysis showed a strong association between SNPs in *TLR2* and *TLR9* and susceptibility to SpA (OR = 12.56; CI = 6.5-25.9 and OR = 1.62; CI = 1.20-2.21, respectively). No association was observed among *HLA-B*^∗^*27* and *TLR* polymorphisms (*p* = 0.72), nor among BASDAI and *TLR* polymorphisms (*p* = 0.85).

**Discussion:**

Our findings suggest that polymorphisms in *TLR2* and *TLR9* genes may contribute to the immunopathogenesis of the SpA. The *rs187084*, *rs5743836*, and *rs5743708* polymorphisms were associated with the risk of SpA development, in this study, and lead to significant changes in the innate and adaptive immune response profile, as well as the maintenance of the regulation of immunological mechanisms.

**Conclusion:**

The polymorphism *rs5743708* for the *TLR2* and the *rs187084_rs5743836 TLR9* haplotypes appear to be involved in the development of clinical forms of SpA and can be a possible therapeutic target for the spondyloarthritis.

## 1. Introduction

Spondyloarthritis (SpA) is a group of rheumatic diseases (RD) with immunological origin that presents chronic inflammatory and autoimmune conditions, and SpA shares clinical, serological, and genetic features, besides presenting a complex pathogenesis [1–3]. This group of diseases includes ankylosing spondylitis (AS), psoriatic arthritis (PsA), reactive arthritis (ReA), arthritis associated with inflammatory bowel disease (IBD), and undifferentiated arthritis (USpA) [1]. AS is the most prevalent clinical form of SpA. In Brazil, 65.1% of the cases of SpA are classified as AS while PsA represents 18.3% [4, 5]. A high number of SpA patients are HLA-B27 positive [1], a molecular marker already associated with AS. In the Brazilian population, the presence of this antigen is around 69.5% [5, 6].

The innate immune response in RD can be stimulated in several cell types through the recognition of molecular patterns from external or internal sources, such as PAMPs or DAMPs (pathogen-associated molecular patterns and damage-associated molecular patterns, respectively) [7–11].

Toll-like receptors (TLRs) are the most well-characterized pattern recognition receptors (PRRs) and are a transmembrane protein coded by the *toll* genes family [7]. TLRs are expressed in different cell types including immune and nonimmune cells; they play a crucial role not only in the detection of many PAMPs and DAMPs but also in the activation and steering of the adaptive immune system by the upregulation of costimulatory molecules of the antigen-presenting cells [4, 7–10].

The endogenous TLR ligand-mediated signaling has an important role in autoimmune disorders [8]. The activation of some TLRs through the interaction of DAMPs can facilitate the repair of damaged tissues and the elimination of cell debris; on the other hand, this same interaction was associated with the chronic inflammatory process involved in RD [12]. Therefore, activation of TLR by DAMPS seems to play a role in the self-sustained inflammatory cycle and the progression of these chronic diseases [7].

The SpA can be considered as multifactorial diseases that have a pathogenesis involving interactions between the environment and genes [2]. The exact mechanisms of SpA immunopathogenesis have not yet been fully elucidated, and probably that other genes outside the MHC also contribute to this complex process.

There is still insufficient data to prove that polymorphisms in the *TLRs* genes are involved in autoimmunity processes and the development of RD [11]. However, some polymorphisms have already been associated with the pathogenesis of RD [4].

In light of this, the purpose of this study was to analyze the influence of important polymorphisms in *TLR2*, *TLR6*, and *TLR9* genes in the immunopathogenesis of the two most common SpA, whereas no study involving these polymorphisms in SpA has been performed in the Brazilian population.

## 2. Materials and Methods

### 2.1. Ethics Statement

This study was approved by the Human Research Ethics Committee of the State University of Maringá—CEP-UEM 687.222/2014, and all volunteer participants signed the informed consent term.

### 2.2. Clinical Characterization

In this case-control study were included 529 subjects living in the south of Brazil. The patient group is composed of 149 unrelated subjects diagnosed with SpA by a single rheumatologist using the ASAS criteria [13], and in patients with PsA we also used the CASPAR criteria [14] to complement the ASAS criteria and thus provide greater security in the data obtained. The patients were attended at the rheumatology outpatient clinic of the University Hospital of Maringá (PR, Brazil), who presented one of the two most frequent clinical forms of SpA in the population (AS or PsA). All patients had magnetic resonance imaging of the sacroiliac joints and were evaluated for the presence of *HLA-B27*.

The control group consists of 380 healthy subjects, and the inclusion criteria in this group were absences of SpA or autoimmune and/or rheumatic disease, unrelated to subjects from the same group or patient group, and residence in the same geographical area as the patients. Both groups were age and sex matched.

### 2.3. Genotyping of TLR Genes

Based on previous published data on the association of *TLR* genes with autoimmune and inflammatory diseases, four SNPs were selected from three *TLR* genes. For the *TLR2* gene (ENSG00000137462), the SNP *rs5743708*: G>A (2258G>A, Arg753Gln); for *TLR6* gene (ENSG00000174130), the SNP *rs5743810*: C>T (745C>T, Ser249Pro); and for *TLR9* gene (ENSG00000239732), the *rs5743836*: T>C (-1237T>C) and *rs187084*: T>C (-1486T>C) SNPs, both located in the promoter region of the *TLR9*. The primer design was according to Folwaczny et al. [15] for the *TLR2* and to Selvaraj et al. [16] for *TLR6* and *TLR9* [15, 16].

The DNA was extracted from the buffy coat by using the salting-out technique [17]. The genotyping of the *TLR* SNPs was performed by polymerase chain reaction and restriction fragment length polymorphism (PCR-RFLP). The PCR was carried out with 100 ng/*μ*L DNA, 200 *μ*M of each dNTP, 0.1 *μ*M of each primer, 1.68 mM of MgCl2, 3 *μ*L 10x PCR buffer, and 1.5 U Taq DNA polymerase (GoTaq® DNA Polymerase, Promega, USA), in a final volume of 15 *μ*L. PCR products were digested using restriction enzymes, temperature, and time of digestion specifically for each SNP (see Table 1).

### 2.4. Genotyping of *HLA-B*^∗^*27*

Genotyping of the *HLA-B*^∗^*27* was performed by PCR-SSP according to Oliveira et al. [18], and a genetic association analysis was performed between the presence or absence of *HLA-B*^∗^*27* and the *TLR* polymorphisms in both clinical forms [18].

### 2.5. Analysis of Disease Activity

Disease activity was assessed by BASDAI (Bath Ankylosing Spondylitis Disease Activity Index), considering patients with high disease activity when BASDAI ≥ 4 [1, 5]. The association analysis was performed among BASDAI and *TLR* polymorphisms.

### 2.6. Ethnic Classification

The ethnic composition of the Brazilian population is influenced by some races [19, 20], which is due to the high ethnic diversity, and considering the Paraná population according to Probst et al. [19], which is of predominantly European origin (80.5%) and has a small but significant contribution of African (12.5%) and Amerindian (7.0%) genes [19, 20].

In this study, the population was considered a mixed ethnic group. Thus, the risk of population stratification bias due to genetic differences presented by different ethnic groups was minimized with a comparison between patients and controls of the same ethnic origin.

### 2.7. Statistical Analysis

The allele and genotype frequencies were estimated and compared by chi-square distribution tables with Fisher's corrections, and the Hardy–Weinberg equilibrium was tested using the Arlequin and SNPStats software [21].

The logistic regression analysis was performed, and the choice of the best inheritance model was performed using the Akaike information (AIC) in order to minimize the expected entropy. Analysis of the linkage disequilibrium (*Δ*′) between the *TLR* SNPs was calculated by the SNPStats software. The EM algorithm or the Markov chain Monte Carlo method was used for the estimation of haplotypes and allelic groups. All tests were performed at a significance level of 5%.

## 3. Results

The mean age for the patient group was 49.5 ± 15 years, composed of 47% men and 53% women (see Table 2). The patients analyzed presented two clinical forms of SpA: AS (95/63.8%) and PsA (54/36.2%) (see Table 3).

The genotypic frequency distribution of all SNPs was in the Hardy–Weinberg equilibrium in both analyzed groups. The codominant model was selected as best inheritance model to analyze the association between SNPs and the risk of developing SpA.

No association was observed among *HLA-B*^∗^*27* and *TLR* polymorphisms (*p* = 0.72), nor among BASDAI and *TLR* polymorphisms (*p* = 0.85). The allele and genotype frequencies of the polymorphisms analyzed in patients and controls (including the two clinical forms) are shown in Table 4.

The variables analyzed in the logistic regression test were gender and age between controls and patients; these results are described below.

The presence of the *rs5743708*^∗^*G/*A for the *TLR2* gene was a susceptibility factor to SpA for males (OR = 12.27, CI = 3.95 − 38.12) and females (OR = 3.56, CI = 1.29 − 9.86), but the risk is higher in men with SpA. However, the polymorphism of the *TLR6* gene was not significantly related to the susceptibility to SpA.

The *rs5743836*^∗^*C* for the *TLR9* gene was associated with susceptibility to SpA (OR = 1.62, CI = 1.20 − 2.21), and the *rs5743836*^∗^*T/C* presented risk for males (OR = 1.84, CI = 1.30 − 3.28), but the *rs5743836*^∗^*C/C* presented risk for females (OR = 9.75, CI = 1.88 − 15.43). Both genotypes associated with risk of SpA development present the *rs5743836*^∗^*C*; however, two copies of this allele seem to only affect women SpA.

The *TLR9 rs187084*^∗^*T/C* was associated with susceptibility to develop SpA only in male patients (OR = 2.51, CI = 1.12 − 5.65).

### 3.1. Association between Haplotypes and Allelic Group in the Development of SpA

We analyzed the haplotypes with frequency ≥ 1% for the *TLR9* gene. The allelic group analyzed in this study was constructed through multiple SNP analyses that included the four SNPs of the investigated *TLR* genes.

The three *TLR9* haplotypes (*rs5743836_ rs187084*) were associated with risk to SpA: the *CC* haplotype (OR = 4.65; CI = 2.44-8.89; *p* < 0.0001); the *CT* (OR = 3.02; CI = 1.62-5.63; *p* = 0.000006); and the *TC* (OR = 1.67; CI = 1.20-2.75, *p* = 0.043), and *CC* haplotype showed linkage disequilibrium (*∆*′ = 0.897, *p* < 0.05). However, the analysis of allelic groups (*rs5743836_ rs187084_rs5743708_rs5743810*) did not show linkage disequilibrium (*∆*′ = 0.1223, *p* > 0.05).

## 4. Discussion

To the best of our knowledge, this is the first study evaluating *TLR2*, *TLR6*, and *TLR9* gene polymorphisms in the immunopathogenesis of SpA. The genetic variability of *TLRs* was featured with an involvement in the susceptibility in inflammatory diseases [7, 22].

Our findings suggest that the polymorphisms analyzed for *Toll-like receptor* genes may contribute to the development of the immunopathogenesis of SpA, without influence of the presence of antigen HLA-B27 or disease activity. The association of other genes in the development of SpA without presence of HLA-B27 antigen was described in the literature by our group, which may indicate that other genetic markers may be involved in SpA [23].

In this present study, the *TLR2* gene *rs5743708*^∗^*A* polymorphism increased the chance of developing SpA by 10-fold. Furthermore, the presence of homozygote (*rs5743708*^∗^*A/A*) was not observed among the controls, confirming our findings that the presence of the altered allele (*A*) may influence the development of SpA. This polymorphism has a very low frequency in the population (<1%) and this allele has been implicated in the risk phenotype [24]. In this way, our data confirm that this polymorphism is a susceptibility factor, also among the spondyloarthritis, in both clinical forms analyzed.

The *TLR2 rs5743708* is a missense variant which affects the structure of the TLR-2 protein in the intracellular region and generates a nonfunctional protein, due to a replacement of an amino acid arginine for glycine at position 753 of the protein (Arg753Gln) [25–27]. This change in TLR-2 reduces the activation of NF-*κ*B pathway and compromises the intracellular signaling cascade [25, 26]. The recognition of PAMPs by these nonfunctional TLR-2 is impaired, leading to failures in the recognition mechanism of the extracellular pathogens, such as the Gram negative/positive bacteria [25, 26]. However, the consequences of this nonfunctional protein are not restricted only to the recognition of pathogens, since TLR-2 activates inflammation through canonical and noncanonical NF-*κ*B pathway [28].

The NF-*κ*B pathway is an important cellular pathway of innate and adaptive immune response, and influences the expression of many genes involved in the regulation of the major processes of activation of the immune response [28]. And the expression of this factor is a major regulator of inflammation and can be active by TLRs (mainly TLR-2 and TLR-9) [28]. There are increasing evidences that suggest a role of NF-*κ*B signaling in the development of various RD; the main genes associated with RD by genome-wide association studies (GWAS) show a correlation with this transcription factor and the production of proinflammatory cytokines [11].

Niebuhr et al. [29] demonstrated a change in the cytokine profile produced by monocytes in patients with the polymorphism *rs5743708* [29]. Levels of IL-12 and IL-6 cytokines were significantly increased, which could explain the increased inflammation in the skin of patients with atopic dermatitis who presented this *TLR2* mutation [29, 30]. Thus, this polymorphism may also affect the profile of the IL-12 cytokine profile in patients with SpA, since high levels of IL- 12 are found in psoriatic lesions and synovial involvement, and the Th17 inflammatory cascade is supported by high levels of IL-12 and IL-6 [31].

Another pattern of cytokines that is modified by the presence of *rs5743708* is the IL-8 profile; as discussed by Nedoszytko and Renke [25], the Th2 response appears to be enhanced by this polymorphism, impairing IL-8 production and may also affect neutrophil adhesion by an increase in IgE levels [25].

The TLR6 polymorphisms suggest that this variation does not present a direct involvement in the development of SpA. However, the frequencies observed for the rs5743810 in this study for patients and controls (21% and 23%) were higher than the world population frequency of 12% [24].

The two analyzed polymorphisms in the *TLR9* gene promoter region have been linked to autoimmunity and gene transcription rate [25, 32–34]. These polymorphisms are regulatory region variants of the *TLR9* gene; the *C* allele of both rs5743836 and *rs187084* polymorphisms is located in the regions of CTF-binding sites and sites of several transcription factors (TF) [24].

The *rs5743836*^∗^*C* creates new NF-*κ*B sites in the promoter region of TLR9 gene [25], and in silico, it has been observed that this allele generates new multiple binding sites for different transcription factors [34]. Furthermore, there is an increase in the expression rate of TLR9 mRNA by stimulation of IL-6, creating a positive feedback loop that amplifies TLR9 signaling through IL-6 in the presence of the *rs5743836*^∗^*T/C* genotype and also interfering in proliferation rate of B cells [34].

Our data suggest that *TLR9 rs5743836*^∗^*C* is a susceptibility factor regardless of gender or age, increasing by 1.69-fold the susceptibility to SpA. In addition, *rs5743836*^∗^*T/C* and *rs5743836*^∗^*C/C* genotypes are an even greater risk factor for patients with PsA. Although there are lacking studies confirming the role of *TLR9 rs5743836* in SpA, there are studies that have previously associated the *rs5743836*^∗^*C* to the development of rheumatoid arthritis (RA) in women [28]. These data corroborate to ours, once that women with SpA, carrier of *rs5743836*^∗^*C*, are at a higher risk than men with *rs5743836*^∗^*C.*

The *rs187084* is associated with increased production of INF-*γ* and TNF-*α* in rs187084^∗^*T/T* subjects, and the *rs187084*^∗^*C* increases the transcriptional rates of *TLR9* [28]. This polymorphism has been associated with susceptibility to diseases, and in the present study a susceptibility was observed only for men with *rs187084*^∗^*T/C*, according to the predisposition already described for men to the development of SpA [28, 35, 36].

Gebura et al. [28] discussed the association among *rs187084*^∗^*T* and increased IFN-*γ* and TNF-*α* productions in RA patients compared to healthy subjects; however, in RA patients, this allele presented a less favorable response to therapies with TNF-*α* inhibitors [28]. Likewise, patients with PsA do not respond to anti-TNF treatment [37]; this polymorphism in the *TLR9* promoter could be influencing cellular and molecular mechanisms in this disease.

In this study, the rs*187084*, *rs5743836*, and *rs5743708* polymorphisms were associated with the risk of SpA development, wherein, according to the literature, these mutations may lead to significant changes in the innate and adaptive immune response profile, as well as in the maintenance of the regulation of immunological mechanisms. In light of these, we may be inferring that these polymorphisms contribute to potentiate the Th1, Th2, and Th17 immune response seen in SpA, which may confer to individuals carrying the polymorphisms a predisposition to the development of SpA.

## 5. Conclusions

Our findings suggest that the polymorphisms analyzed for *Toll-like receptor* genes may contribute to the development of the immunopathogenesis of SpA, independently of the presence of antigen HLA-B27.

The polymorphisms analyzed for *Toll-like receptors* genes may contribute to the development of the immunopathogenesis of ankylosing spondylitis and psoriatic arthritis. The polymorphisms *rs5743708* for the *TLR2* and the *rs187084_rs5743836 TLR9* haplotypes appear to be involved in the development of clinical forms of PsA and can be a possible therapeutic target for the rheumatic diseases. However, further studies are needed to more clearly understand the influence of the immunogenetics of these polymorphisms in the development of SpA, and these observations should be interpreted with caution due to limitations found in this study, such as the relatively small sample size and the fact that there were no analyses of the expression of the *TLR* genes nor of the cytokines involved in the SpA development process.

## Figures and Tables

**Table 1:** Primer sequences, restriction enzymes used, and restriction digestion patterns for genotyping of *TLRs* genes.

Gene	SNP	Primer sequences	Restriction enzymes	Restriction temperature (°C) (time)	Length of the restriction fragments
*TLR2*	rs5743708	F: 5′-CATTCCCCAGCGCTTCTGCAAGCTCC-3′	Msp I	37°C (overnight)	Allele G—104 bp + 25 bp
		R: 5′-GGAACCTAGGACTTTATCGCAGCTC-3′			Allele A—210 bp

*TLR6*	rs5743810	F: 5′-GCATTTCCAAGTCGTTTCTATGT- 3′	Ava II	37°C (3 hours)	Allele C—50 bp + 160 bp
		R: 5′-GCAAAAACCCTTCACCTTGTT- 3′			Allele T—210 bp

*TLR9*	rs5743836	F: 5′-CTGCTTGCAGTTGACTGTGT -3′	Mva I	37°C (3 hours)	Allele C—27 bp + 48 bp + 60 bp
		R: 5′-ATGGGAGCAGAGACATAATGGA-3′			Allele T—34 bp + 111 bp

	rs187084	F: 5′-TATCGTCTTATTCCCCTGCTGGAATGT-3′	Afl II	37°C (1 hour)	Allele T—34 bp + 111 bp
		R: 5′-TGCCCAGAGCTGACTGCTGG-3′			Allele C—145 bp

F: forward standard; R: reverse standard; TLR: toll-like receptor; °C: degrees Celsius; bp: base pair.

**Table 2:** Demographic data in control and SpA patients for the analyzed SNPs.

	*TLR2 rs5743708*		*TLR6 rs5743810*		*TLR9 rs5743836*		*TLR9 rs187084*	
	Controls *n* = 380	Patients *n* = 149	Controls *n* = 221	Patients *n* = 149	Controls *n* = 380	Patients *n* = 149	Controls *n* = 221	Patients *n* = 149
Age (years) ± mean	58.4 ± 15.4	49.5 ± 15	48.9 ± 13.7	49.5 ± 15	58.4 ± 15.4	49.5 ± 15	48.9 ± 13.7	49.5 ± 15
Gender (*n* as %)								
Female	200 (53)	78 (53)	113 (51.3)	78 (53)	200 (53)	78 (53)	113 (51.3)	78 (53)
Male	180 (47)	71 (47)	108 (48.7)	71 (47)	180 (47)	71 (47)	108 (48.7)	71 (47)

**Table 3:** Clinical characteristics of patient groups.

	SpA *n* = 149	AS *n* = 95	PsA *n* = 54
Female/male (*n* as %)	79/70 (53.0/47.0)	51/44 (52.7/47.3)	28/26 (51.8/48.1)
Family history SpA (*n* as %)	39 (58.1)	9 (13.4)	30 (44.7)
BASDAI ≥ 4 (*n* as %)	104 (69.8)	69 (73.4)	35 (65.5)
Disease duration-years (mean ± SD)	11.6 ± 8.6	10.8 ± 8	13.2 ± 10
Treatment time-years (mean ± SD)	4 ± 3.7	7.8 ± 7.9	6.3 ± 5.9
HLA-B^∗^27 (*n* as %)			
Present	83 (55.7)	53 (55.8)	30 (55.5)
Absent	66 (44.3)	42 (44.2)	24 (44.5)

SpA: patients with spondyloarthritis; AS: ankylosing spondylitis; PsA: psoriatic arthritis; BASDAI: Bath Ankylosing Spondylitis Disease Activity Index.

**Table 4:** Allelic and genotypic frequencies for the *TLR2* gene *rs5743708*, the *TLR6 rs5743810*, and *TLR9 rs5743836* and *rs187084* among the control and patient groups.

	Controls	Patients					
		SpA	OR (95% CI)^∗^^a^	AS (%)	OR (95% CI)^∗^^b^	PsA (%)	OR (95% CI)^∗^^c^
*TLR2 rs5743708*	*n* = 380	*n* = 149		*n* = 95		*n* = 54	
Genotype							
G/G	369 (97.1)	112 (75.2)		72 (76)		40 (74)	
G/A	11 (2.9)	31 (20.8)	9.28 (4.52-19.07)	19 (20)	10.06 (4.17-24.28)	12 (22)	8.99 (4.09-19.73)
A/A	0	6 (4)	19.68 (2.86-166)	4 (4)	20.73 (2.41-179.4)	2 (4)	25.42 (3.46-223.2)
Allele							
G	374 (99)	127 (85.2)	0.098 (0.03-0.24)	80 (84.3)	0.099 (0.034-0.27)	46 (85)	0.093 (0.031-0.27)
A	6 (1)	22 (14.8)	10.52 (4.07-31.83)	13 (13.7)	10.05 (3.76-29.5)	8 (15)	10.73 (3.6-32.63)

*TLR6 rs5743810*	*n* = 221	*n* = 149		*n* = 95		*n* = 54	
Genotype							
C/C	135 (61.1)	93 (62.4)	n.s	56 (59)	n.s	36 (67.7)	n.s
C/T	77 (34.8)	46 (30.9)	n.s	32 (33.7)	n.s	14 (26)	n.s
T/T	9 (4.1)	10 (6.7)	n.s	7 (7.3)	n.s	4 (7.3)	n.s
Allele							
C	174 (79)	115 (77)	n.s	73 (76.8)	n.s	43 (79.7)	n.s
T	46 (21)	34 (23)	n.s	22 (23.2)	n.s	11 (20.3)	n.s

*TLR9 rs5743836*	*n* = 380	*n* = 149		*n* = 95		*n* = 54	
Genotype							
T/T	244 (64.2)	73 (49)		52 (54.7)		23 (42.6)	
T/C	128 (33.7)	68 (45.6)	1.77 (1.19-2.62)	39 (41.1)	n.s	27 (50)	2.33 (1.28-4.25)
C/C	8 (2.1)	8 (5.4)	3.37 (1.22-9.31)	4 (4.2)	1.60 (1.01-2.54)	4 (7.4)	5.52 (1.54-19.81)
Allele							
T	308 (81.1)	107 (71.8)	0.59 (0.38-0.92)	70 (73.7)	n.s	37 (68.5)	n.s
C	72 (18.9)	42 (28.2)	1.69 (1.62-2.62)	25 (26.3)	n.s	17 (31.5)	n.s

*TLR9 rs187084*	*n* = 221	*n* = 149		*n* = 95		*n* = 54	
Genotype							
T/T	86 (39)	64 (42.9)	n.s	46 (48.4)	n.s	18 (33.4)	n.s
T/C	103 (46.5)	68 (45.7)	n.s	41 (43.2)	n.s	26 (48.1)	n.s
C/C	32 (14.5)	17 (11.4)	n.s	8 (8.4)	n.s	10 (18.5)	n.s
Allele							
T	137 (62)	98 (65.8)	n.s	68 (71.6)	n.s	31 (57.4)	n.s
C	84 (38)	51 (34.2)	n.s	27 (28.4)	n.s	23 (42.6)	n.s

*n*: number of individuals; SpA: patients with spondyloarthritis; AS: ankylosing spondylitis; PsA: psoriatic arthritis; OR: odds ratio; CI: confidence interval with *p* < 0.05; n.s: not significant. ^∗^Calculated using the chi-square test; ^a^SpA *vs* controls; ^b^AS *vs* controls; ^c^PsA *vs* controls.

## Data Availability

The authors declare that all the data that support the results of this study are available in the article.

## References

[1] Sieper J., Rudwaleit M., Khan M. A., Braun J. (2006). Concepts and epidemiology of spondyloarthritis. *Best Practice & Research Clinical Rheumatology*.

[2] Lipton S., Deodhar A. (2012). The new ASAS classification criteria for axial and peripheral spondyloarthritis: promises and pitfalls. *International Journal of Clinical Rheumatology*.

[3] Taurog J. D., Chhabra A., Colbert R. A. (2016). Ankylosing spondylitis and axial spondyloarthritis. *The New England Journal of Medicine*.

[4] Brentano F., Kyburz D., Schorr O., Gay R., Gay S. (2005). The role of toll-like receptor signalling in the pathogenesis of arthritis. *Cellular Immunology*.

[5] Skare T. L., Bortoluzzo A. B., Gonçalves C. R. (2012). Ethnic influence in clinical and functional measures of Brazilian patients with spondyloarthritis. *The Journal of Rheumatology*.

[6] Sampaio-barros P. D., Azevedo V. F., Bonfiglioli R., Campos W. R. (2007). Consenso Brasileiro de Espondiloartropatias : Outras Espondiloartropatias Diagnóstico e Tratamento – Primeira Revisão First update on the Brazilian Consensus for the Diagnosis and Treatment of Spondyloarthropathies : other spondyloarthropathies. *Revista Brasileira de Reumatologia*.

[7] Joosten L. A. B., Abdollahi-roodsaz S., Dinarello C. A., Neill L. O., Netea M. G. (2016). Toll-like receptors and chronic inflammation in rheumatic diseases: new developments. *Nature Reviews Rheumatology*.

[8] Ropert C. (2019). How toll-like receptors reveal monocyte plasticity: the cutting edge of antiinflammatory therapy. *Cellular and Molecular Life Sciences*.

[9] Vidya M. K., Kumar V. G., Sejian V., Bagath M., Krishnan G., Bhatta R. (2018). Toll-like receptors: significance, ligands, signaling pathways, and functions in mammals. *International Reviews of Immunology*.

[10] Vijay K. (2018). Toll-like receptors in immunity and inflammatory diseases: Past, present, and future. *International Immunopharmacology*.

[11] Kato M. (2018). The role of genetics and epigenetics in rheumatic diseases: are they really a target to be aimed at?. *Rheumatology International*.

[12] Arida A., Protogerou A. D., Kitas G. D., Sfikakis P. P. (2018). Systemic inflammatory response and atherosclerosis: the paradigm of chronic inflammatory rheumatic diseases. *International Journal of Molecular Sciences*.

[13] Rudwaleit M., Landewé R., van der Heijde D. (2009). The development of Assessment of SpondyloArthritis international Society classification criteria for axial spondyloarthritis (part I): classification of paper patients by expert opinion including uncertainty appraisal. *Annals of the Rheumatic Diseases*.

[14] Chandran V., Schentag C. T., Gladman D. D. (2008). Sensitivity and specificity of the CASPAR criteria for psoriatic arthritis in a family medicine clinic setting. *The Journal of Rheumatology*.

[15] Folwaczny M., Glas J., Török H., Limbersky O., Poliklinik C. F. (2004). Toll‐like receptor (TLR) 2 and 4 mutations in periodontal disease. *Clinical and Experimental Immunology*.

[16] Selvaraj P., Harishankar M., Singh B., Jawahar M. S., Banurekha V. V. (2010). Toll-like receptor and TIRAP gene polymorphisms in pulmonary tuberculosis patients of South India. *Tuberculosis*.

[17] Cardozo D. M., Guelsin G. A., Clementino S. L. (2009). Extração de DNA a partir de sangue humano coagulado para aplicação nas técnicas de genotipagem de antígenos leucocitários humanos e de receptores semelhantes à imunoglobulina DNA extraction from coagulated human blood for application in genotyping techniq. *Revista da Sociedade Brasileira de Medicina Tropical*.

[18] Oliveira G. C., Ambrosio-Albuqerque E., Visentainer J. E. L. (2016). Application of PCR-SSP method for *HLA-B*^∗^*27* identification as an auxiliary tool for diagnosis of ankylosing spondylitis. *Jornal Brasileiro de Patologia e Medicina Laboratorial*.

[19] Probst A. C. M., Bompeixe E. P., Pereira N. E. (2014). HLA polymorphism and evaluation of European, African, and Amerindian contribution to the White and Mulatto populations from Paraná, Brazil. *Human Biology*.

[20] Parra F. C., Amado R. C., Lambertucci J. R., Rocha J., Antunes C. M., Pena S. D. (2003). Color and genomic ancestry in Brazilians. *Proceedings of the National Academy of Sciences of the United States of America*.

[21] Solé X., Guinó E., Valls J., Iniesta R., Moreno V. (2006). SNPStats: a web tool for the analysis of association studies. *Bioinformatics*.

[22] Netea M. G., Wijmenga C., O'Neill L. A. J. (2012). Genetic variation in Toll-like receptors and disease susceptibility. *Nature Immunology*.

[23] Loures M. A. R., Macedo L. C., Reis D. M. (2018). Influence of *TNF* and *IL17* gene polymorphisms on the spondyloarthritis immunopathogenesis, regardless of HLA-B27, in a Brazilian population. *Mediators of Inflammation*.

[24] Kersey P. J., Allen J. E., Allot A. (2018). Ensembl Genomes 2018: an integrated omics infrastructure for non-vertebrate species. *Nucleic Acids Research*.

[25] Nedoszytko B., Lange M., Renke J. (2018). The possible role of gene variant coding nonfunctional toll-like receptor 2 in the pathogenesis of Mastocytosis. *International Archives of Allergy and Immunology*.

[26] Xiong Y., Song C., Snyder G. A., Sundberg E. J., Medvedev A. E. (2012). R753Q polymorphism inhibits Toll-like receptor (TLR) 2 tyrosine phosphorylation, dimerization with TLR6, and recruitment of myeloid differentiation primary response protein 88. *The Journal of Biological Chemistry*.

[27] Gao Y., Xiao H., Wang Y., Xu F. (2017). Association of single-nucleotide polymorphisms in toll-like receptor 2 gene with asthma susceptibility. *Medicine*.

[28] Gębura K., Świerkot J., Wysoczańska B. (2017). Polymorphisms within genes involved in regulation of the nf-*κ*b pathway in patients with rheumatoid arthritis. *International Journal of Molecular Sciences*.

[29] Niebuhr M., Langnickel J., Draing C., Renz H., Kapp A., Werfel T. (2008). Dysregulation of toll‐like receptor‐2 (TLR‐2)‐induced effects in monocytes from patients with atopic dermatitis: impact of the TLR‐2 R753Q polymorphism. *Allergy*.

[30] Mrabet-Dahbi S., Dalpke A. H., Niebuhr M. (2008). The Toll-like receptor 2 R753Q mutation modifies cytokine production and Toll- like receptor expression in atopic dermatitis. *The Journal of Allergy and Clinical Immunology*.

[31] Thibodaux R. J., Triche M. W., Espinoza L. R. (2018). Ustekinumab for the treatment of psoriasis and psoriatic arthritis: a drug evaluation and literature review. *Expert Opinion on Biological Therapy*.

[32] Lazarus R., Klimecki W. T., Raby B. A. (2003). Single-nucleotide polymorphisms in the Toll-like receptor 9 gene (*TLR9*): frequencies, pairwise linkage disequilibrium, and haplotypes in three U. S. ethnic groups and exploratory case – control disease association studies. *Genomics*.

[33] Onalan E., Halit E., Salih E. (2011). The investigation of toll-like receptor 3, 9 and 10 gene polymorphisms in Turkish rheumatoid arthritis patients. *Rheumatology International*.

[34] Carvalho A., Osório N. S., Saraiva M. (2011). The C allele of rs5743836 polymorphism in the human TLR9 promoter links IL-6 and TLR9 up-regulation and confers increased B-cell proliferation. *PLoS One*.

[35] Pray C., Irene N. I., Haroon N. N. (2017). Bone mineral density and fracture risk in ankylosing spondylitis: a meta-analysis. *Calcified Tissue International*.

[36] Lin H., Gong Y. (2017). Association of HLA-B27 with ankylosing spondylitis and clinical features of the HLA-B27-associated ankylosing spondylitis: a meta-analysis. *Rheumatology International*.

[37] Wu D., Yue J., Tam L. (2018). Efficacy and safety of biologics targeting interleukin-6, -12/23 and -17 pathways for peripheral psoriatic arthritis: a network meta-analysis. *Rheumatology*.

